# Interpreting Infrared Thermography with Deep Learning to Assess the Mortality Risk of Critically Ill Patients at Risk of Hypoperfusion

**DOI:** 10.31083/j.rcm2401007

**Published:** 2023-01-04

**Authors:** Jing-chao Luo, Huan Wang, Shang-qing Tong, Jia-dong Zhang, Ming-hao Luo, Qin-yu Zhao, Yi-jie Zhang, Ji-yang Zhang, Fei Gao, Guo-wei Tu, Zhe Luo

**Affiliations:** ^1^Department of Critical Care Medicine, Zhongshan Hospital, Fudan University, 200032 Shanghai, China; ^2^Hybrid Imaging System Laboratory, Shanghai Engineering Research Center of Intelligent Vision and Imaging, School of Information Science and Technology, ShanghaiTech University, 201210 Shanghai, China; ^3^Shanghai Medical College, Fudan University, 200032 Shanghai, China; ^4^College of Engineering and Computer Science, Australian National University, Canberra, ACT 2601, Australia; ^5^Department of information and intelligence development, Zhongshan Hospital, Fudan University, 200032 Shanghai, China; ^6^Department of Critical Care Medicine, Xiamen Branch, Zhongshan Hospital, Fudan University, 361015 Xiamen, Fujian, China

**Keywords:** deep learning, infrared thermography, hypoperfusion, critically ill patients, secondary analysis

## Abstract

**Background:**

Hypoperfusion, a common manifestation of many critical illnesses, could lead to abnormalities in body surface thermal distribution. However, the interpretation of thermal images is difficult. Our aim was to assess the mortality risk of critically ill patients at risk of hypoperfusion in a prospective cohort by infrared thermography combined with deep learning methods.

**Methods:**

This post-hoc study was based on a cohort at high-risk of hypoperfusion. Patients’ legs were selected as the region of interest. Thermal images and conventional hypoperfusion parameters were collected. Six deep learning models were attempted to derive the risk of mortality (range: 0 to 100%) for each patient. The area under the receiver operating characteristic curve (AUROC) was used to evaluate predictive accuracy.

**Results:**

Fifty-five hospital deaths occurred in a cohort consisting of 373 patients. The conventional hypoperfusion (capillary refill time and diastolic blood pressure) and thermal (low temperature area rate and standard deviation) parameters demonstrated similar predictive accuracies for hospital mortality (AUROC 0.73 and 0.77). The deep learning methods, especially the ResNet (18), could further improve the accuracy. The AUROC of ResNet (18) was 0.94 with a sensitivity of 84% and a specificity of 91% when using a cutoff of 36%. ResNet (18) presented a significantly increasing trend in the risk of mortality in patients with normotension (13 [7 to 26]), hypotension (18 [8 to 32]) and shock (28 [14 to 62]).

**Conclusions:**

Interpreting infrared thermography with deep learning enables accurate and non-invasive assessment of the severity of patients at risk of hypoperfusion.

## 1. Introduction

Tissue hypoperfusion is a common manifestation of many critical illnesses and is also one of the major contributing factors of in-hospital death [1, 2, 3]. For patients at risk of hypoperfusion, clinicians should recognize the relevant risk factors, assess the current severity, take necessary interventions, and monitor consequent changes. In recent years, investigators have been exploring methods, which are easy to be used and non-physician dependent, to assess the severity of patients with hypoperfusion. These tools, such as lactate, skin mottling and capillary refill time (CRT) [4], however, are difficult to reconcile simplicity with accuracy.

Physiologically, the continuity and quantity of skin blood flow is reduced when tissue perfusion deteriorates, which in turn results in uneven thermal distribution on the body surface [5]. Several studies have found that surface temperature differences and trajectories correlate with the prognosis of patients with sepsis [6, 7]. Our group have established a prospective cohort gathering infrared images of the legs of critically ill patients at high risk of hypoperfusion, collecting routine hypoperfusion parameters and following up on their prognosis. Based on these data, we defined parameters reflecting thermal inhomogeneity of body surface (e.g., low temperature area rate [LTAR] and standard deviation [SD]) using traditional mathematical methods and found that these parameters varied among patients with different severity of hypoperfusion and could be used to predict the risk of mortality [8].

However, the accuracy of interpreting body surface infrared images based on conventional algorithms to predict mortality risk is not yet very satisfactory. As we have known, the body surface thermal distribution is visually a two-dimensional grey-scale image and, in principle, deep learning algorithms (especially convolutional neural networks), which are excellent at supervised image recognition tasks, can identify and interpret the information behind these thermal images [9] and thus enabling more accurate prediction of the severity for patients at high risk of hypoperfusion.

We performed this post hoc analysis of the cohort dataset with the aim of developing deep learning models to interpret infrared thermography to assess the mortality risk of patients at risk of hypoperfusion.

## 2. Materials and Methods

### 2.1 Patients

This study conducted a post-hoc analysis of a 373-patient cohort at risk of hypoperfusion from a cardiac surgical intensive care unit (ICU) during a one-year period (June 2020 to May 2021) [8]. This cohort was established with the approval of the Ethics Committee of Zhongshan Hospital, Fudan University (Number B2020-057). Patients with any high-risk factors of hypoperfusion were enrolled, including hypotension, cardiac dysfunction, tachycardia, hyperlactatemia, oliguria, skin mottling or prolonged CRT [8]. The exclusion criteria were patients <18 years, pregnant, severe arterial or cutaneous abnormalities, other conditions impeding acquisition of complete image or expected to be transferred out of ICU within 24 hours [8].

### 2.2 Data Collection

Data for this study were obtained from body surface infrared images of the original cohort [8]. Thermal images of patient’s legs (below the perineum and above the ankle) were acquired by an infrared thermography (A615, 640 × 480 pixels, ±0.05 °C, Teledyne FLIR LLC, CA, USA) in the supine position and then converting to temperature matrix by using the official FLIR tools software. Background values outside the lower limb area were removed. With the thermal data, several parameters of thermal inhomogeneity (SD, Kurtosis [10], Skewness [10], Entropy [11] and LTAR) were calculated. The LTAR was defined as the proportion of the leg area with a temperature lower than 10% of the maximum temperature [8]. Demographics, routine laboratory examinations, conventional circulatory and hypoperfusion parameters were collected. The dose of vasopressor or inotropes was transferred to vasoactive inotropic score (VIS) [12].

### 2.3 Outcome Definitions

The circulatory status was divided into three categories, i.e., normotension, hypotension (systolic blood pressure [SBP] <90 mmHg or vasopressor use) or shock (hypotension and hyperlactatemia [lactate ≥4 mmol/L]) [13], for comparisons among subgroups. All patients were followed up until death or hospital discharge and the hospital mortality was used as the primary outcome.

### 2.4 Statistical Analysis

**Fig. 1.**
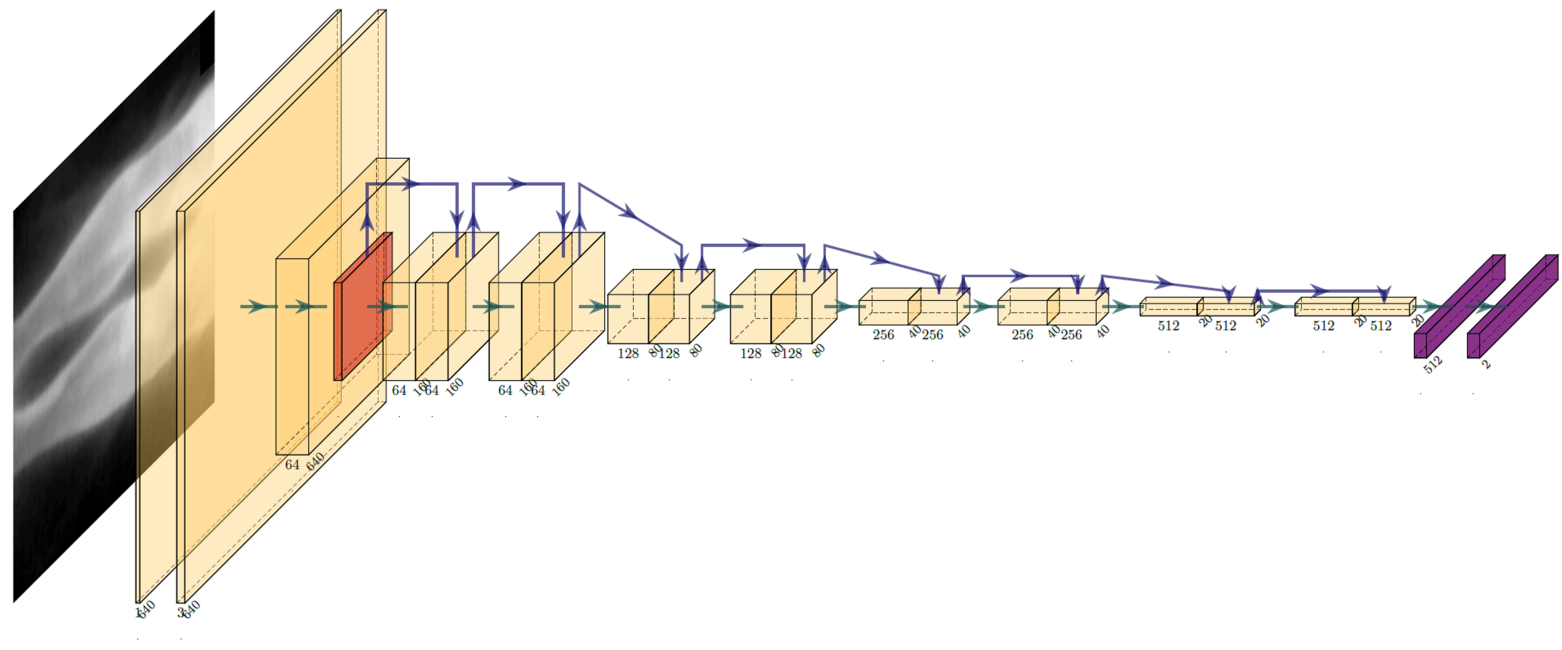
**Construction of a predictive model based on convolutional neural networks**.

Data were presented as the mean ± SD (if normal) or median with interquartile range (IQR) (if non-normal) or total numbers with percentage and compared with Student’s *t*-test or Wilcoxon (or Friedman) rank-sum test or Fisher’s exact test, as appropriate. The temperature matrix of lower limbs were used as input. Deep learning models were constructed using convolutional neural network frameworks. Depending on the backbone and the number of layers, there were six models: Alexnet [14], Mobilenet v3 [15], Shufflenet v2 (1.0 or 1.5) [16], Resnet (18 or 34) [17] (Fig. 1 & **Supplementary Fig. 1**). The final outputs of the models were the risk of mortality, ranging from 0–100%.

Receiver operating characteristic (ROC) curves were generated and the areas under the ROC curves (AUROC) were calculated to evaluate the predictive accuracy for mortality risk. Sensitivity, specificity, positive and negative predictive values (PPV and NPV) and associated 95% confidence intervals (CI) were calculated based on the cutoff value as determined by the Youden Index. The gray zone of best cutoff and patients in the gray zone was also calculated [18]. Calibration plot and Brier score were used to assess the agreement between predictions and observations. In addition, we used a 5-fold cross-validation to assess internal validity. The relationships among model outputs and conventional variables were explored by two-dimensional histograms with Loess regression curves. Statistical analyses were performed using Python (version 3.9, Python Software Foundation, Delaware, USA) and R (version 4.1.1, R Foundation for Statistical Computing,Vienna, Austria), *p *< 0.05 was considered statistically significant.

## 3. Results

### 3.1 Characteristics of Study Cohort

Of the 373 patients, 55 patients died during hospital stay. The median length of hospital stay was 16 [IQR 12–105] days. When compared to the surviving patients, the deceased patients had higher heart rate (HR: 100 *vs.* 89 bpm, *p <* 0.001) and VIS (23 *vs.* 6 μg/kg/min, *p *< 0.001) while lower diastolic blood pressure (DBP: 50 *vs.* 59 mmHg, *p <* 0.001), mean arterial pressure (MAP: 67 *vs.* 75 mmHg, *p <* 0.001). Besides, hematological (Hemoglobin: 8.2 *vs.* 9.5 g/dL, *p <* 0.001; Platelet: 80 *vs.* 107 × ${\mathrm{10}}^{9}$/L, *p *< 0.001), renal (Creatinine: 172 *vs.* 115 μmol/L, *p *< 0.001), hepatic (Bilirubin: 44 *vs.* 23, *p *< 0.001), cardiac (N-terminal pro brain natriuretic peptide: 8099 *vs.* 1384 pg/mL, *p <* 0.001) and infectious (Procalcitonin: 7.7 *vs.* 1.0,* p *< 0.001) parameters were also worse in dead patients. There were also significant differences in the urine output (UO) (1.3 *vs.* 0.8 mL/kg/h, *p *< 0.001), lactate (1.9 *vs.* 7.0 mmol/L, *p *< 0.001), CRT (1.1 *vs.* 1.9, *p *< 0.001) and occurrence of skin mottling (1% *vs.* 20%, *p <* 0.001) between surviving and deceased patients.

### 3.2 Performance of Deep Learning Models

**Fig. 2.**
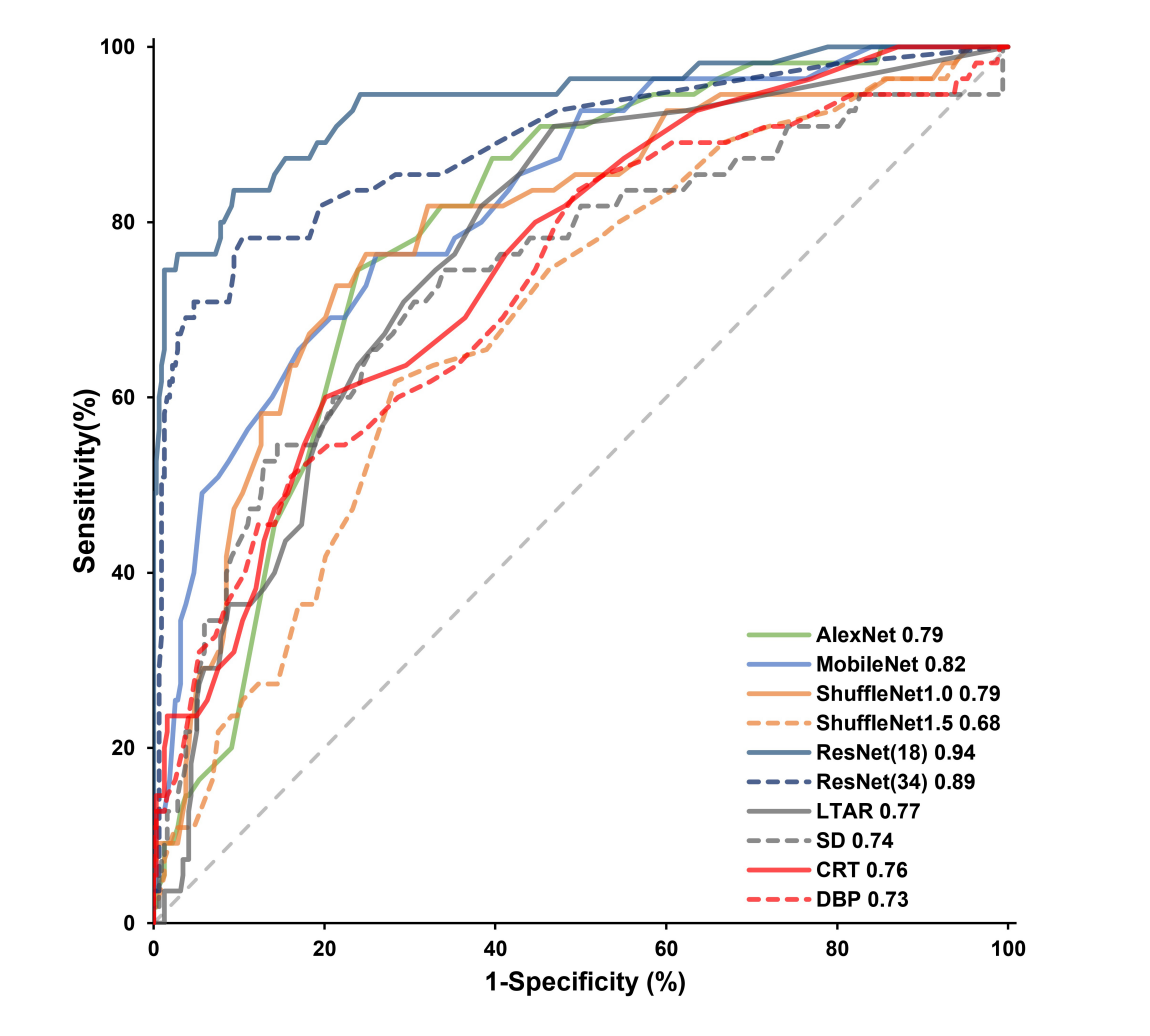
**Receiver operating characteristic curves for deep learning models and conventional hypoperfusion or thermal parameters**.

The AUROCs of six deep learning models for predicting hospital mortality ranged from 0.68 to 0.94 (Table 1 and Fig. 2). Of them, ResNet (18) (AUROC: 0.94 [95% CI: 0.91–0.96]) and ResNet (34) (AUROC: 0.89 [95% CI: 0.85–0.92]) had better predictive accuracy than other models. The ResNet (34) model, despite more neural network layers and larger multiply-accumulate operations than ResNet (18), did not further improve the prediction accuracy. Therefore, we finally settled on the ResNet (18) model as the final choice. The best cutoff value of ResNet (18) was 36% and only 58 (13%) patients located in the gray zone of 27% to 38%. Correspondingly, the sensitivity, specificity, PPV and NPV were 84 (95% CI: 71–92), 91 (95% CI: 87–94), 58 (95% CI: 46–68) and 97 (95% CI: 94–99), respectively.

**Table 1.** **Predictive accuracies of deep learning models and hypoperfusion parameters**.

Models or parameters	AUROC	Best cutoff	Patients in gray zone	Sensitivity	Specificity	PPV	NPV
AlexNet, %	0.79 (0.75–0.83)	38 (32–42)	137 (37%)	75 (61–85)	76 (71–81)	33 (25– 41)	95 (91–97)
MobileNet, %	0.82 (0.78–0.86)	34 (26–40)	144 (39%)	76 (63–87)	74 (69–79)	32 (24–41)	95 (91–97)
ShuffleNet1.0, %	0.79 (0.75–0.83)	39 (25–50)	182 (49%)	76 (63–87)	75 (70–80)	34 (25–43)	95 (91–97)
ShuffleNet1.5, %	0.68 (0.64–0.73)	32 (25–41)	232 (62%)	62 (48–75)	72 (66–77)	25 (18–33)	91 (87– 95)
ResNet (18), %	0.94 (0.91–0.96)	36 (27–38)	48 (13%)	84 (71–92)	91 (87–94)	58 (46–68)	97 (94–99)
ResNet (34), %	0.89 (0.85–0.92)	16 (3–17)	129 (35%)	78 (65–88)	90 (86–93)	54 (43–66)	96 (93–98)
LTAR, %	0.77 (0.72–0.81)	3 (2–16)	143 (38%)	83 (71–93)	62 (56–67)	26 (20–33)	96 (92–98)
SD, °C	0.74 (0.69–0.78)	0.87 (0.63–1.17)	230 (62%)	76 (62–87)	66 (61–72)	27 (20–35)	94 (90–96)
CRT, s	0.76 (0.71–0.80)	1.7 (1.0–2.3)	201 (54%)	61 (47–74)	80 (75–84)	30 (22–40)	92 (88–95)
DBP, mmHg	0.73 (0.68–0.77)	48 (45–64)	215 (58%)	52 (38–66)	84 (80–88)	36 (25–49)	90 (86–93)

CRT, capillary refill time; LTAR, low temperature area rate; SD, standard deviation; DBP, diastolic blood pressure.

### 3.3 Models Validation

**Supplementary Fig. 2** shows the calibration curves for each deep learning model. ResNet (18) had the best calibration curve performance and the lowest Brier score at 4.8, followed by ResNet (34), while several other models had much worse calibration curve performance. In the cross-validation (**Supplementary Fig. 3**), the AUROCs of the deep learning models all had fluctuations, but their average values were still relatively consistent with the values in Table 1. Of these, ResNet (18) has the most consistent performance in terms of folds.

### 3.4 Comparisons with Conventional Parameters

According to our previous study [8], CRT (AUROC: 0.76 [95% CI: 0.71–0.80]), DBP (AUROC: 0.73 [95% CI: 0.68–0.77]) and LTAR (AUROC: 0.77 [95% CI: 0.72–0.81]), SD (0.74 [95% CI: 0.69–0.78]) were the most accurate predictors for risk of mortality among conventional circulatory and body surface thermal parameters. In comparison, the AUROC of ResNet (18) is significantly better than all of these parameters. The grey areas for conventional parameters were also much wider (Table 1), which means that a significant number of patients would be misclassified. Thus, we also found that the PPVs of the conventional parameters were indeed much lower than that of ResNet (18).

### 3.5 Mortality Risk Derived from Deep Learning Model in Different Perfusion Status

For subgroups with normotension, hypotension and shock, they had decreased mean arterial pressure (78 to 70 to 65 mmHg) and urine output (1.3 to 1.2 to 0.8 mL/kg/h, *p *< 0.001) and increased lactate (1.8 to 1.9 to 7.0), Δ${\mathrm{PCO}}_{2}$ (6.9 to 7.8 to 8.9), CRT (1.1 to 1.3 to 1.6 s) and occurrence of skin mottling (2 to 2 to 16%), but there was no significant differences in ${\mathrm{ScvO}}_{2}$ (71 *vs.* 69 *vs.* 69%, *p *= 0.225). For the thermal inhomogeneity parameters, LTAR increased from 1 to 3 to 7%, while SD increased from 0.81 to 0.88 to 0.94 °C. In addition, we found that the risk of mortality derived from deep learning models also exhibited increasing trends in normotension, hypotension and shock patients (Table 2). For example, the mortality probability given by Resnet (18) steadily increases from 13 to 18 to 28% (Table 2).

**Table 2.** **Distribution of mortality risks derived from deep learning methods in different perfusion status**.

	Normotension (n = 195)	Hypotension (n = 127)	Shock (n = 51)	*p* value
Alexnet, %	29 (21 to 39)	32 (25 to 41)	37 (30 to 41)	<0.001
Mobilenet, %	25 (18 to 37.50)	28 (21 to 37)	34 (25.50 to 44)	0.001
Shufflenet1.0, %	29 (21 to 40)	30 (20 to 44.50)	39 (27.50 to 54)	0.001
Shufflenet1.5, %	27 (24 to 33)	31 (25 to 37)	32 (29 to 39)	<0.001
Resnet (18), %	13 (7 to 26)	18 (8 to 32)	28 (14 to 62)	<0.001
Resnet (34), %	2 (1 to 8)	3 (1 to 9.50)	12 (3 to 71)	<0.001

### 3.6 Mortality Risk Derived from Deep Learning Model Correlated with Conventional Parameters

**Fig. 3.**
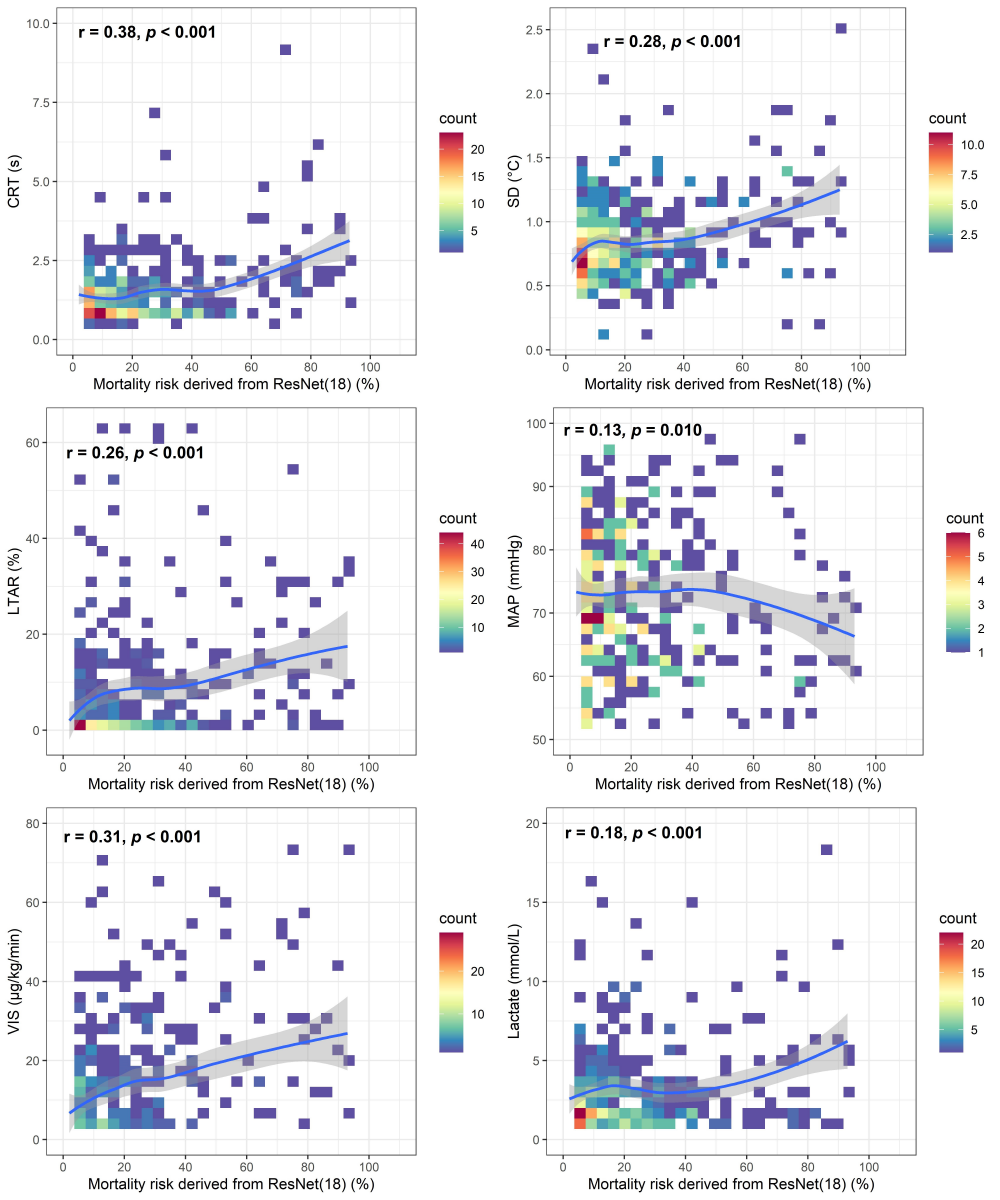
**Two-dimension histograms with loess regression curves between ResNet (18) deep learning model and conventional hypoperfusion or thermal parameters**.

There were general correlations between the output of the deep learning model, i.e., the risk of mortality, and parameters of conventional circulation, hypoperfusion and thermal inhomogeneity. As the risk of mortality increases, patient’s perfusion pressure (MAP) gradually decreased and parameters reflecting the severity of hypoperfusion (CRT, VIS, lactate) gradually increased, along with thermal inhomogeneity parameters (LTAR and SD) (Fig. 3).

## 4. Discussion

This study was conducted with a cohort dataset of critically ill patients at risk of hypoperfusion. Deep learning algorithms were developed to interpret the information contained in the infrared thermographic images of the patients’ legs. Of them, the model based on the residual network had superior accuracy in predicting mortality risk and demonstrated general correlations with conventional perfusion parameters and the severity of hypoperfusion.

Medical scientists have long noted that local changes in blood flow or metabolism can lead to thermal abnormalities, which can then be used to diagnose diseases, such as breast cancer [19, 20] and arterial stenosis [21]. Such an approach focusing on changing in thermal parameters has been expanded to the intensive care units recently. Peripheral to central temperature gradient was found to be correlated with perfusion pressure and cardiac output [5]. Besides, toe-to-room temperature gradient could reflect the severity of sepsis [6]. Nagori *et al*. [22] also used deep learning to interpret whole-body infrared images to achieve prediction of the probability of shock in pediatric patients.

Combing infrared thermography and deep learning algorithms to study hypoperfusion has great potential in making more accurate predictions of patient’s mortality risk. Traditionally, CRT is a single, non-invasive, easily accessible, and most prognostically relevant parameter of hypoperfusion. Our previous work showed that infrared thermography-based parameters of inhomogeneity in body surface thermal distribution, such as LTAR and SD, had similar accuracies to CRT and could achieve higher predictive precision when used in combination (AUROC: 0.865) [8].

Despite good interpretability, the accuracy of algorithms constructed on the basis of conventional methods has reached a ceiling and there is little potential for further improvement. Considering that the body surface thermal distribution is a two-dimensional grey-scale image, we can apply deep learning algorithms, particularly convolutional neural networks [9, 23, 24], to exploit the information behind these images and thus make more accurate predictions about patients’ risk of mortality.

In this study, six models were constructed using a deep learning framework based on convolutional neural networks. These models also varied in accuracy, complexity and the amount of computation required to process each sample. AlexNet introduces a Rectified Linear Unit (ReLU) function with a simpler architecture and faster training, achieving an accuracy (AUROC: 0.79) similar to that of CRT and LTAR in this study population. MobileNet, with its hardware-aware network architecture search, could realize higher accuracy (AUROC: 0.82) in a relatively short time. ShuffleNet has a much lighter architecture. We tried the second version, which is currently the most mainstream, but its accuracy improvement was not significant (AUROC: 0.68 and 0.79). ResNet adopted shortcut connections within every stage, so that the stacked layers learn the residual information. The ResNet (18) model is well balanced, with high accuracy (AUROC: 0.89 and 0.94) and moderate model complexity and requirements of computing power. In the near future, we propose to create an online tool to help other healthcare providers to use our models.

Our study has several limitations. Firstly, this post-hoc study was based on data from a single center. In this dataset, temperature distribution data were measured only once per patient. In the future, validation using external data has also been planned. Secondly, only one image was taken per patient. Dynamic monitoring would be more helpful for clinical management. Thirdly, some patients are excluded because of lower limb vascular disease or pregnancy, reducing the applicability of the population to some extent. Foutrh, the infrared thermography which was not calibrated with a blackbody had better sensitivity than accuracy. Finally, the present study was based primarily on the cardiac critical illness population. The accuracy of the prediction model in other critically ill populations, particularly sepsis, needs to be validated.

## 5. Conclusions

The interpretation of infrared thermography images using deep learning algorithms enables non-invasive and more accurate assessment of the risk of mortality in critically ill patients at risk of hypoperfusion.

## Data Availability

The datasets used and/or analyzed during the current study are available from the corresponding author on reasonable request.

## Abbreviations

CRT, capillary refill time; LTAR, low temperature area rate; SD, standard deviation; ICU, intensive care unit; SBP, systolic blood pressure; ROC, receiver operating characteristic; PPV, positive predictive value; NPV, negative predictive value; CI, confidence interval; UO, urine output; VIS, vasoactive inotropic score.

## Supplementary Material

Supplementary material associated with this article can be found, in the online version, at https://doi.org/10.31083/j.rcm2401007.
