# Supplementary material for: Interpreting Infrared Thermography with Deep Learning to Assess the Mortality Risk of Critically Ill Patients at Risk of Hypoperfusion
Source: Rev Cardiovasc Med. 2023 Jan 4;24(1):7. doi: 10.31083/j.rcm2401007 (PMC11270443; doi:10.31083/j.rcm2401007)
Supplement: Supplementary file 1 [file 2153-8174-24-1-007-s1.zip › Supplementary Material.docx]

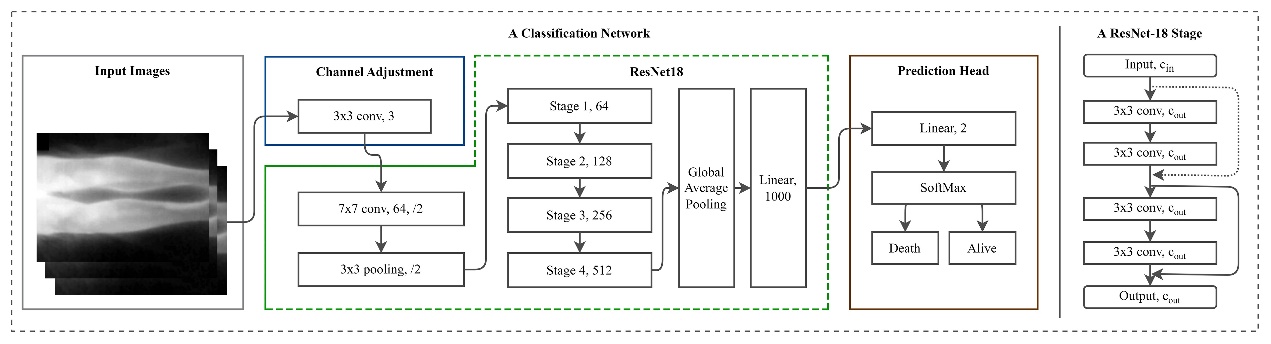


Supplementary Fig. 1. An example network architecture for the classification task.

Left: our implemented ResNet-18 model as a reference. Right: the architecture of a single stage in ResNet-18. The dotted connection serves as a shortcut connection, which is an identity operation when c_in=c_out while an 1×1 convolution to match number of channels when c_in≠c_out.


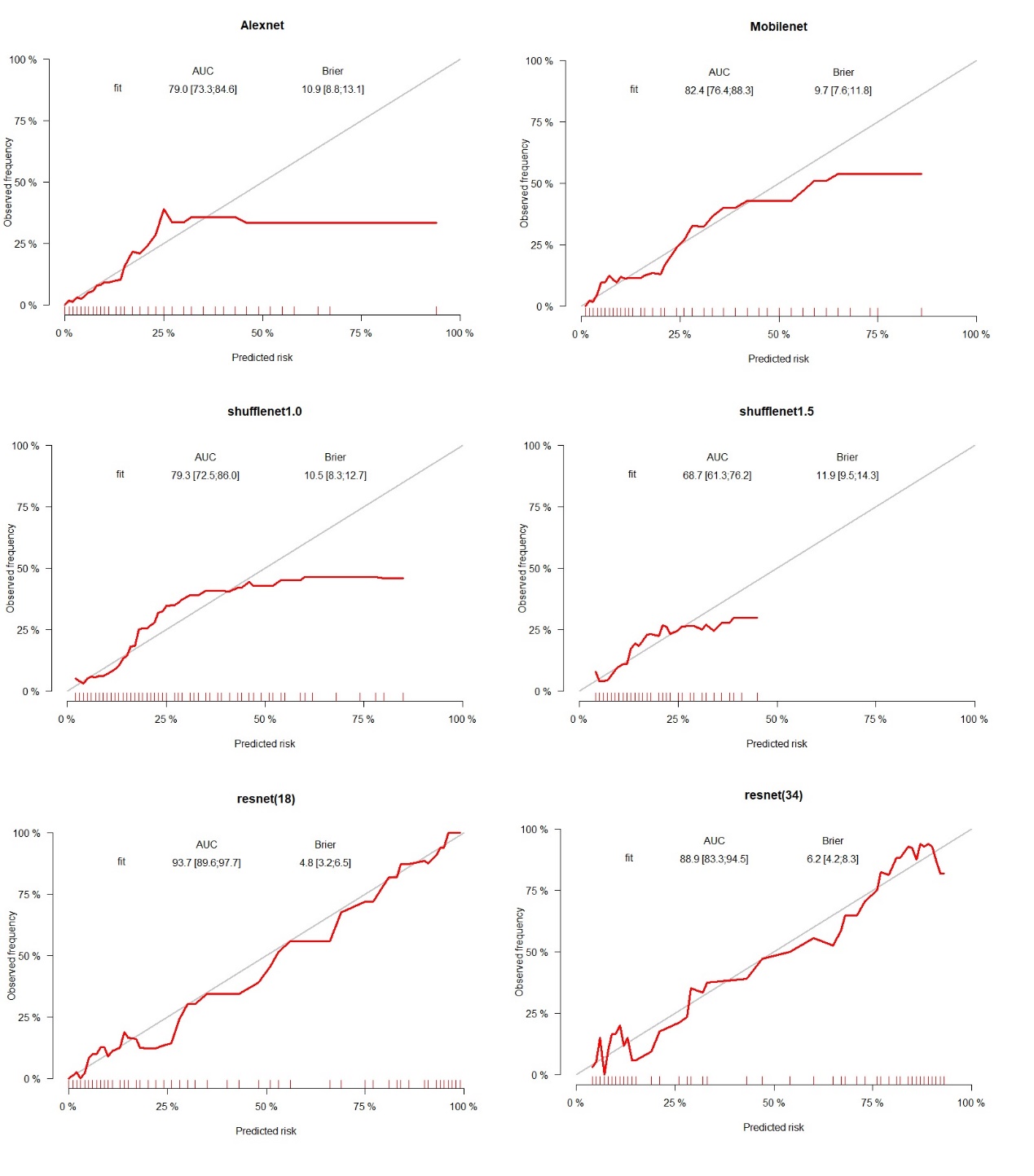


Supplementary Fig. 2. Calibration curves


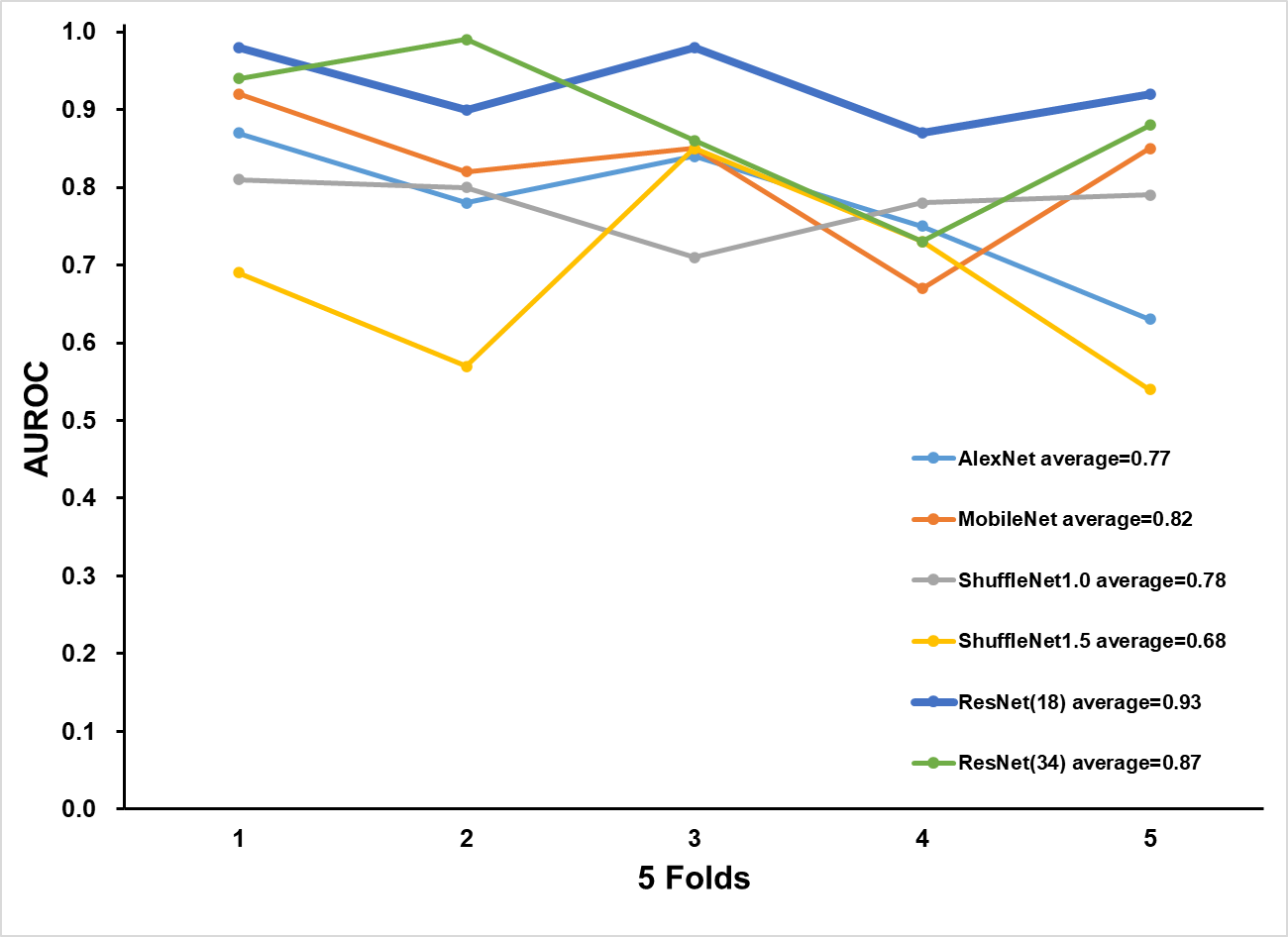


Supplementary Fig. 3. 5-fold cross validation
