# Supplementary figures and images for: Interpreting Infrared Thermography with Deep Learning to Assess the Mortality Risk of Critically Ill Patients at Risk of Hypoperfusion
Source: Rev Cardiovasc Med. 2023 Jan 4;24(1):7. doi: 10.31083/j.rcm2401007 (PMC11270443; doi:10.31083/j.rcm2401007)

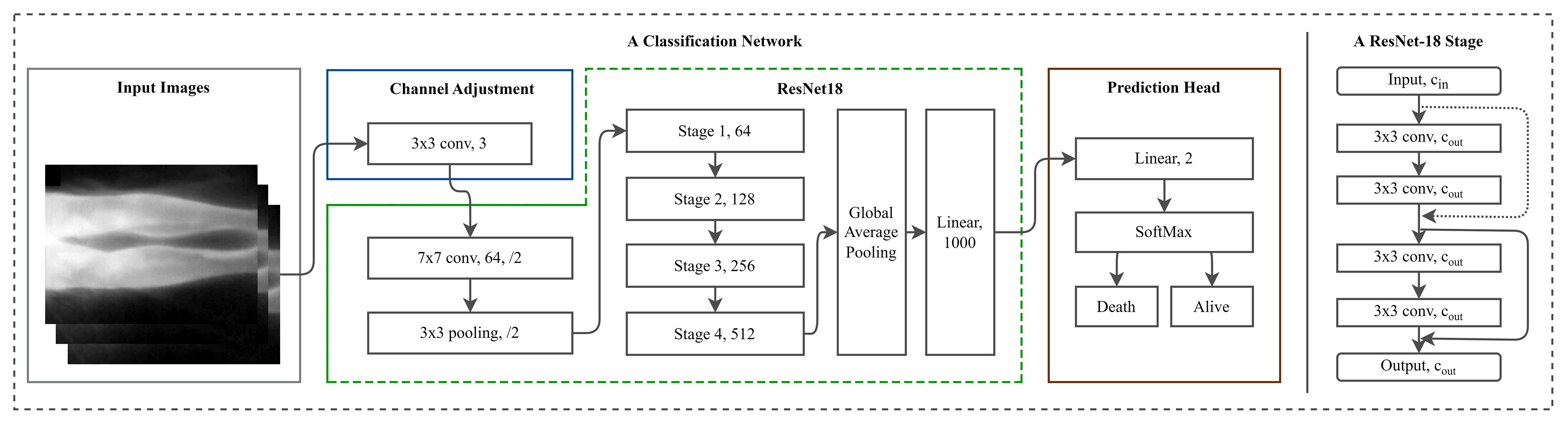

Supplement: Supplementary file 1 [file 2153-8174-24-1-007-s1.zip › Supplementary Fig. 1.jpg]

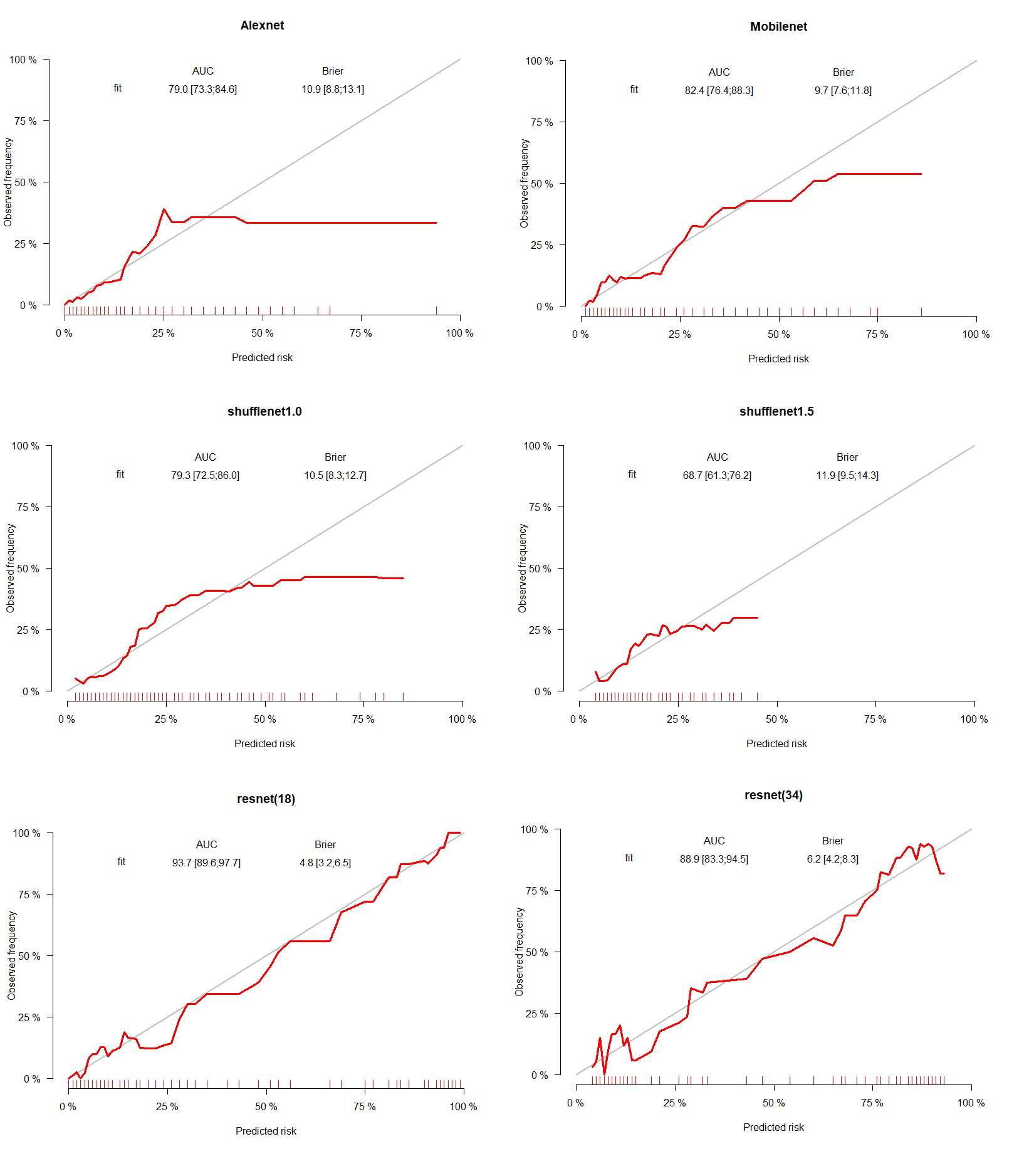

Supplement: Supplementary file 1 [file 2153-8174-24-1-007-s1.zip › Supplementary Fig. 2.jpg]

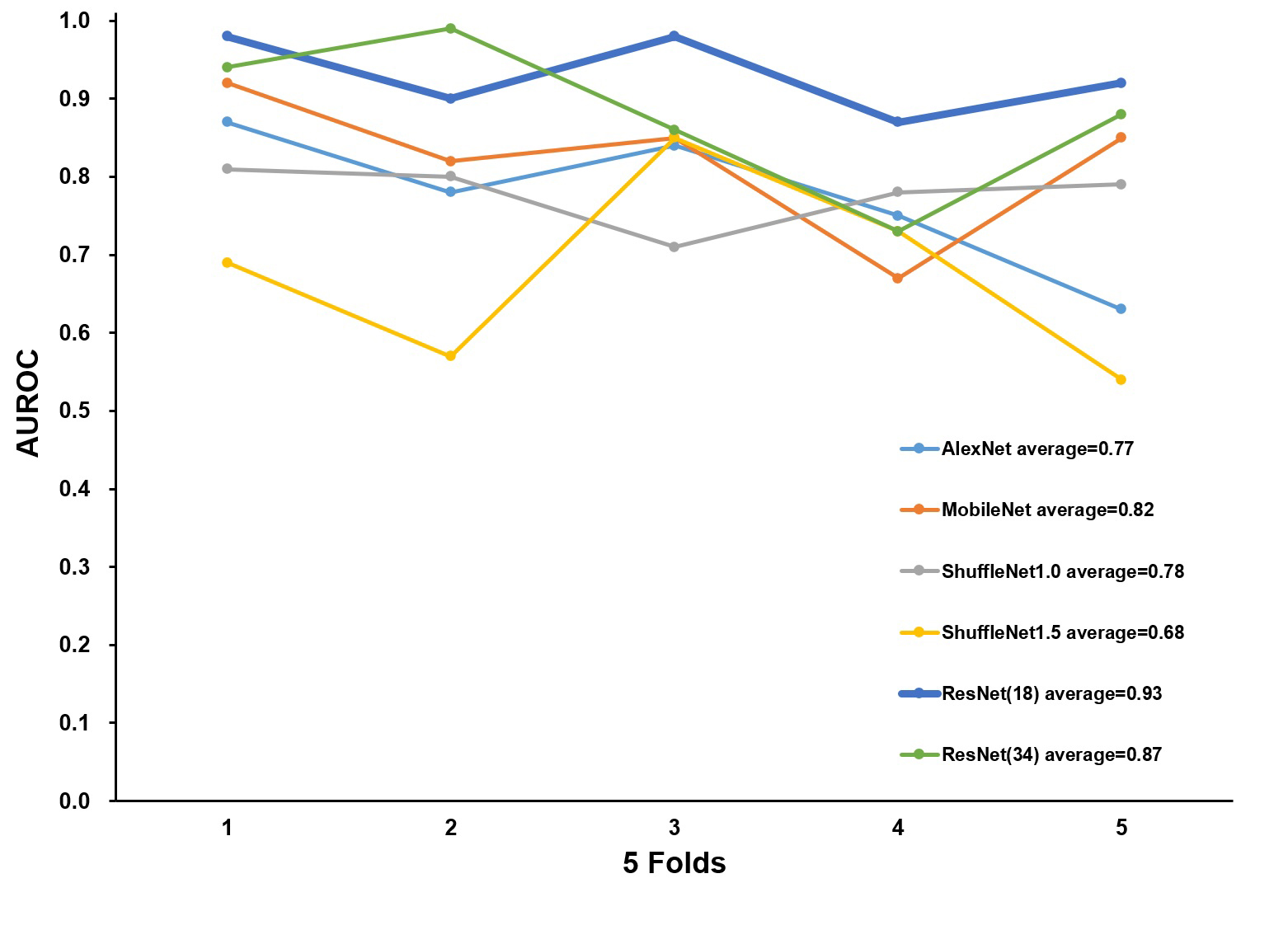

Supplement: Supplementary file 1 [file 2153-8174-24-1-007-s1.zip › Supplementary Fig. 3.jpg]
